# Measuring melasma patients' quality of life using willingness to pay and time trade-off methods in thai population

**DOI:** 10.1186/1471-5945-11-16

**Published:** 2011-12-19

**Authors:** Charussri Leeyaphan, Rungsima Wanitphakdeedecha, Woraphong Manuskiatti, Kanokvalai Kulthanan

**Affiliations:** 1 Department of Dermatology, Faculty of Medicine Siriraj Hospital, Mahidol University, Bangkok, THAILAND

## Abstract

**Background:**

Melasma is a common hyperpigmentation disorder that has a significant effect on an individual's quality of life. However, there is no preference-based measurement that reflects quality of life in patients with melasma. The objective of this study was to assess the impact of melasma on quality of life by using a health status measurement - the Dermatology Life Quality Index (DLQI) - and a preference-based measurement - Willingness to Pay (WTP) and Time Trade-Off (TTO).

**Methods:**

A cross-sectional descriptive study was conducted. Seventy-eight patients with melasma who attended the melasma clinic at Siriraj Hospital from February to March 2009 were recruited in this study. The Thai version of the DLQI, questionnaires about WTP, standard TTO, and daily TTO were used to assess patients' quality of life.

**Results:**

Seventy-seven (98.7%) patients were female with a mean age of 47.8 ± 7.9 years. The mean health utility based on standard TTO was 0.96. The utility obtained by the daily TTO method was 0.92 and was significantly correlated with an economically inactive occupation (p < 0.05). The mean monthly WTP for the most effective treatment was 1,157 baht (7.2% of monthly income), ranging from 100 to 5,000 baht (1 USD ~ 35.1 baht). The WTP was significantly correlated with monthly personal income and the total DLQI score.

**Conclusion:**

The WTP method could be a useful tool with which to measure the quality of life of patients with melasma.

## Background

Melasma is a common hyperpigmentation disorder that occurs on sun-exposed areas of the skin, most commonly involving the cheeks, nose, forehead, and chin. Melasma affects more non-Caucasians than Caucasians [1-3]. In 2007, 1,169 melasma patients attended Siriraj Hospital (4.1% of all dermatological outpatients). Melasma usually begins with brownish macules and progresses to patches with well-defined borders. The brownish patches have no surface scale and have a symmetric distribution [4]. Melasma can have a significant effect on an individual's quality of life as it usually affects the face and causes disfiguring lesions [5]. The Melasma Area Severity Index (MASI) is used by physicians to evaluate the severity of melasma; however, this index does not indicate the effect of melasma on the patients' quality of life [6,7]. Dermatologists should incorporate health-related quality of life measurements to help assess and monitor the progression of their patients [8,9]. Treatment of melasma is challenging because of the chronic and persistent nature of the condition [10].

Health status measurement is well established and widely used, capturing a detailed image of the impact of dermatological conditions on different dimensions of quality of life. The limitation of this instrument is its potential insensitivity to the overall impact of the disease on quality of life [8,11]. The Dermatology Life Quality Index (DLQI) is a generic health status measurement for all dermatological diseases. This questionnaire contains general questions and may not be specific enough to detect important aspects of a particular disease [11]. The English version of the DLQI was translated to Thai for use in Thai people with skin diseases. This version has proven high validity and good reliability [12]. The Melasma Quality of Life (MQoL) is a disease-specific health status measurement. Previous studies demonstrated that the discriminatory ability of MQoL is superior to that of the DLQI index [9,13]. The MQoL scores were highly correlated with DLQI and MASI scores [13]. The Thai version of the MQoL has not been tested for validity and reliability and thus cannot be used to evaluate Thai melasma patients.

A preference-based measurement is an assessment method that is based on economic theory, allowing patients to theoretically give up something of value (money, time, risk of death) in order not to have the disease in question. Thus, this method of assessment provides an insight into the burden of disease as it relates to quality of life [8,11]. There are two types of preference-based measurement, including utility measurement and contingent evaluation. The most widely used direct utility measurement is Time Trade-Off (TTO), in which patients exchange a proportion of their future survival time in exchange for perfect health or for the most effective treatment during a shortened life span [11,14]. Another way that investigators have attempted to capture patient preferences in dermatology is through daily TTO. This method was modified by asking patients to allocate time (hours per day) for imaginary therapy [11,15]. Many dermatological studies have used this method in patients with psoriasis,[8,16,17] atopic dermatitis,[8,16] and acne [16]. The Willingness To Pay method (WTP) is a contingent evaluation requiring respondents to imagine a market for a program or health benefit and to reveal the maximum that they would be willing to pay for that program or benefit. A higher WTP indicates a worse quality of life and thus, as with utilities, WTP is a measure of the quality of life disease burden. WTP has been used for patients with psoriasis [8,15,18-20], port wine stains [21], atopic dermatitis [8,22] and acne [23]. In Thailand, the cost of melasma treatment is not covered by any payment scheme. The WTP for cosmetic dermatological procedures promises to become an increasingly relevant issue as the prevalence of these procedures, which by definition require out-of-pocket expenditure, continues to increase [24]. Preference-based quality of life measurements (WTP and TTO) have the advantage that they provide easy to compare numerical values across different disease states and demonstrate the detrimental effects of a disease on quality of life to those who manage resource allocation and funding. They are appropriate measures for incorporating quality of life into pharmaco-economic valuations [11]. However, there have not been any studies on preference-based measurements in melasma patients.

The primary objective of the present study was to measure the health state utilities of patients with melasma attending the melasma clinic at a university-based hospital by using health status (DLQI) and preference-based (TTO, WTP) measurement. A secondary aim was to determine the correlation between preference-based (TTO, WTP) measurements, the health status measurement (DLQI), clinical, and socioeconomic factors.

## Methods

### Patients and design of the study

This study was approved by the ethics committee on research involving human subjects at Siriraj Hospital, Bangkok, Thailand. It is a cross-sectional descriptive study based on a questionnaire. The study population comprised patients older than 18 years old, with clinically diagnosed melasma who had attended the melasma clinic at the dermatology outpatient service at the Faculty of Medicine, Siriraj Hospital, Mahidol University, from February to March 2009. All study subjects were contacted on a visit to the clinic and invited to participate in the study. Informed consent was obtained from all subjects participated in this study.

### Intervention

All interviews were conducted by two physicians at the clinic. The questionnaires consisted of three parts: a self-administered questionnaire that included socio-demographic questions, questions about melasma and current treatments, questions about heath status measurement (DLQI), and preference-based measurements (TTO, WTP). The patients were also examined by the interviewer in order to assess the MASI score.

The Thai version of the DLQI measures how much a skin problem has affected the patient's life over the previous 7 days. It consists of 10 items. Each question has five possible answers: "not related", "not at all", "a little", "a lot", and "very much", which correspond to scores of 0, 0, 1, 2, and 3, respectively. The overall summary score ranges between 0 (the best score) and 30 (the worst score) [12].

The WTP is assessed by a closed-ended questionnaire. The bidding approach was used to determine the maximum WTP. Data in the questions adapt from studying of the combination of topical medication that is the most effective but has the least side effects in a period [25]. For example, "Suppose the doctor prescribes the most effective topical medication that is applied once daily for 8 weeks resulting in complete clearing in 30% of treated patients and significant improvement in 70% of treated patients for 1 year, but has a 1% incidence of side effects, how much would you be willing to pay for this medication?" The answer is the number of baht per treatment (8 weeks) divided by 2 to find the number of baht per month.

The TTO is assessed by questionnaire. Data in the questions adapt from studying of the combination of topical medication that is the most effective but has the least side effects in a period [25]. There are two types of questions, including:

1. Standard TTO: According to 2008 WHO data, the life expectancy of Thai males and females is 69 and 75 years, respectively. The average age of melasma patients is about 40 years [5] and most are female; thus patients are expected to live for about another 30 years. In this study, the question asked was "Imagine, you (patients) will live for another 30 years with melasma. If the doctor prescribed the most effective topical medication that should be applied once daily for 8 weeks resulting in complete clearing in 30% of patients and significant clearing in 70% of patients for 1 year, but has a 1% incidence of side effects, how many years would you exchange for this medication?" The answer is "A" years per treatment. The utility obtained by the standard TTO were 30-A/30.

2. Daily TTO: The question was "Imagine, if the doctor prescribed the most effective topical medication that should be applied once daily for 8 weeks resulting in complete clearing in 30% of patients and significant improvement in 70% of patients, but has a 1% incidence of side effects, how many hours per day would you exchange for this medication?" The answer is "B" hours per treatment. The utility obtained by the daily TTO were 24-B/24.

### Statistical method

Descriptive analysis was used to describe the frequency, mean, standard deviation, and 95% confidence interval (CI) of clinical, socioeconomic, DLQI, WTP, and TTO data to define the impact on quality of life in melasma patients.

The Spearman correlation was used to test correlation between different methods. Multivariate regression analysis was carried out, using ordinary least squares, to examine the relationship between preference-based measurements (WTP, TTO) and disease characteristics, and socioeconomic data such as monthly income, age, education, and occupation. All statistical tests were carried out with a 5% level of significance for two-sided tests. All statistical data analyses were performed using SPSS for Windows version 10.

## Results

A total of 78 patients (mean age 47.8 years, range 28-66) completed the study questionnaire and the interview. All patients were clinically diagnosed as having melasma by a dermatologist. Demographic, socioeconomic and clinical data are shown in Table 1 and 2.

**Table 1:** Demographic and clinical data of patients with melasma.

Characteristics	No. of melasma patients (n = 78)
Mean age ± SD (years)	47.8 ± 7.9
Sex:	
- Female - Male	77(98.7%) 1 (1.3%)
Marriage status:	
- Married - Single - Divorced	51 (65.4%) 24 (30.8%) 3 (3.8%)
Education:	
- Bachelor's degree - Secondary level - Elementary level - Vocational training - Master's degree - Doctor's degree	33 (42.3%) 17 (21.8%) 13 (16.7%) 10 (12.8%) 4 (5.1%) 1 (1.3%)
Occupation:	
- Housewife - Private employee - Government employee - Laborer - Officer - Nurses	25 (32.1%) 17 (21.8%) 16 (20.5%) 13 (16.7%) 5 (6.4%) 2 (2.6%)
Mean monthly personal income ± SD	23,391 ± 26,138 baht (666.4 ± 744.7$)

**Table 2:** Melasma history and clinical data of melasma patients.

Characteristics	No. of melasma patients (n = 78)
Mean age of onset of melasma ± SD (years)	38.3 ± 9.2
Predisposing factors for melasma	
- Sun exposure - Oral contraceptive pill - Pregnancy - Sun-exposure due to occupation - Sun-exposure due to sport	73 (93.6%) 11 (14.1%) 10 (12.8%) 9 (11.5%) 4 (5.1%)
Have family members with melasma	42 (53.8%)
The mean score on disease activity (MASI)	14.8 ranging from 1.2-42
The main topical treatment patients received	
- Hydroquinone - Sunscreen - Retinoic acid - Azalaic acid - Steroid	74 (94.9%) 71 (91%) 65 (83.3%) 56 (71.8%) 41 (53.6%)
Mean duration of treatment ± SD (years)	5.5 ± 6.2

The mean score of the DLQI was 7.3. Question 2, which questioned about embarrassment, displayed the highest mean score. (Table 3) The reliability of this DLQI in this study was demonstrated by Cronbach's alpha. It was 0.709 which represented homogeneity of the study population.

**Table 3:** Percentage of responses to each question of the DLQI in melasma patients.

Question	Percentage					Median DLQI
		
	Not at all (0)		A lot (2)	Very much (3)	Not relevant (0)	
1. Itchy/sore/painful/stinging skin	76.9	16.7	5.1	1.3	0	0
2. Embarrassment	25.6	21.9	26.9	25.6	0	2
3. Shopping/home	28.2	24.4	21.7	24.4	1.3	1
4. Clothes	61.4	24.4	10.3	2.6	1.3	0
5. Social activities	28.2	20.4	24.4	24.4	2.6	1
6. Sport	50	15.4	17.9	16.7	0	0.5
7. Working or studying	84	4.5	5.1	0	6.4	0
8. Interpersonal problems	59	11.5	12.8	1.3	15.4	0
9. Sexual difficulties	69.2	3.8	2.6	0	24.4	0
10. Treatment difficulties	67.9	21.8	6.4	0	3.9	0

The mean health utility based on the standard TTO was 0.96. Multivariate analysis demonstrated that sex, age, education, occupation, income, marital status, duration of treatment and MASI score were not significantly correlated with utilities measured by the standard TTO (p > 0.05). The detail of analysis including dependent and independent variables are shown in Table 4.

**Table 4:** Multivariate analysis between quality of life from standard TTO method and demographic- socioeconomic-clinical data was estimated by ordinary least square methods.

Variable	Coefficient	t-Statistic	p-value
C	0.912	9.44	0.000
AGE	1.78E-05	0.009	0.993
EDU	0.034	1.030	0.307
OCC	0.025	0.706	0.483
MASI	0.000	0.251	0.803
TR	0.001	0.525	0.601
MS	0.013	0.387	0.700
INC	1.40E-07	0.238	0.813

Dependent Variable: TTY.

R square: 0.028 Adjusted R square: -0.069

F-statistic: 0.288, p-value 0.956

Where:

TTY: Quality of life obtaining from standard TTO method

AGE: Age of patient (years)

EDU: Dummy variable which 1 if education level is bachelor or higher level and 0 otherwise

OCC: Dummy variable which 1 = economic inactive occupation (housewife, pensioner, unemployed) and 0 = otherwise

MASI: Score of MASI

TR: Duration of treatment (years)

MS: Dummy variable which 1 if marital status = married and 0 = otherwise

INC: Monthly personal income (Baht)

On average, patients were willing to offer 1.9 ± 1.4 hours per day for the most effective treatment. The utility obtained by the daily TTO method was 0.92. An economically inactive occupation, such as housewife, was significantly (p < 0.05) correlated with utility obtained by the daily TTO. In other word, a melasma patient's quality of life was significantly lower if they had an economically inactive occupation. However, sex, age, education, income, marital status, duration of treatment and MASI score were not significantly correlated with utility measured by the daily TTO. The regression analysis with stepwise method demonstrated the value of R^2 ^and F statistic more than the regression with enter method. Criteria of stepwise method was entering when probability of F was ≤ 0.05 and removing when probability of F was ≥ 0.10. The summary model had only occupation as independent variable. It excluded variable of age, education, MASI score, married status, income and duration of treatment. The correlation between quality of life measuring by daily TTO and demographic-socioeconomic-clinical factors was shown in Table 5.

**Table 5:** Multivariate analysis between quality of life measuring by daily TTO method and demographic-socioeconomic-clinical factors.

Variable	Coefficient	t-Statistic	p-value
C	0.931	121.955	0.000
OCC	-0.035	-2.629	0.010*

Dependent variable: TTD.

R square: 0.083 Adjusted R square: 0.071

F-statistic: 6.910, p-value 0.010

* Significant values (p-value < 0.05)

Where:

TTD: Quality of life obtaining from daily TTO method

OCC: Dummy variable which 1 = economic inactive occupation (housewife, pensioner, unemployed) and 0 = otherwise.

The mean monthly WTP for the most effective treatment in this period was 1,157 baht (33 USD), ranging from 100 to 5,000 baht (2.8 USD-142.5 USD). Income was significantly (p < 0.05) correlated with the WTP in a regression analysis controlling for other clinical-socioeconomic data. An increase in monthly personal income of 100 baht increased the WTP by 2 baht. Age, sex, education, occupation, marital status, duration of treatment and MASI score were not significantly correlated with mean monthly WTP. The correlation between monthly WTP and demographic-socioeconomic-clinical factors were estimated by multivariate regression. The regression with stepwise method had the value of R2 and F-statistic more than the regression with enter method, so this study used regression with stepwise method. The summary model had income as independent variable. It excluded variable of age, occupation, education, MASI score, married status and duration of treatment. The correlation between monthly WTP and demographic-socioeconomic-clinical factors were shown in Table 6.

**Table 6:** Multivariate analysis between monthly WTP and demographic-socioeconomic-clinical factors.

Variable	Coefficient	t-Statistic	p-value
C	596.201	4.320	0.000*
INC	0.024	6.073	0.000*

Dependent Variable: MWTP.

R square: 0.327 Adjusted R square: 0.318

F-statistic: 36.877, p-value 0.000

* Significant values (p-value < 0.05)

Where:

MWTP: Monthly WTP (Baht)

INC: Monthly personal income (Baht).

The mean monthly WTP was 7.2% of monthly personal income. Multivariate analysis demonstrated that the WTP in terms of relative monthly income was significantly correlated with occupation and age (p < 0.05). Melasma patients' quality of life was significantly lower if patients had an economically inactive occupation or were younger. Sex, education, marital status, duration of treatment and MASI score were not significantly correlated with mean monthly WTP. The correlation between impact on quality of life measuring by WTP(WTP/income) and demographic-socioeconomic-clinical factors estimated by multivariate regression. The regression with stepwise method had the value of R2 and F-statistic more than the regression with enter method, so this study used regression with stepwise method. The summary model had age and occupation as independent variable. It excluded variable of education, MASI score, married status and duration of treatment. The correlation between impact on quality of life measuring by WTP and demographic-socioeconomic-clinical factors were shown in Table 7.

**Table 7:** Multivariate analysis between impact on quality of life from WTP/income method and demographic-clinical-socio-economic factors.

Variable	Coefficient	t-Statistic	p-value
C	0.158	3.686	0.000*
AGE	-0.002	-2.301	0.024*
OCC	0.044	2.902	0.005*

Dependent Variable: WTP/income.

R square: 0.125 Adjusted R square: 0.102

F-statistic: 5.380, p-value 0.007

* Significant values (p-value < 0.05)

Where:

WTP/income: Impact on quality of life obtaining from WTP method (monthly WTP/monthly income)

AGE: Age of patient (years)

OCC: Dummy variable which 1 = economic inactive occupation (housewife, pensioner, unemployed) and 0 = otherwise.

The Spearman correlations between the total DLQI score and the patients' quality of life measuring by standard TTO, daily TTO and WTP were shown in Table 8.

**Table 8:** Spearman correlation coefficient between quality of life according to different methods of measurement.

Correlation		DLQI	Standard TTO	Daily TTO	WTP/INC	WTP
DLQI	Coefficient	1	0.107	-0.176	0.240	0.226
	p value	-	0.352	0.122	**0.035**	**0.047**
Standard TTO	Coefficient		1	0.266	0.009	0.150
	p value		-	**0.019**	0.936	0.191
Daily TTO	Coefficient			1	-0.134	0.087
	p value			-	0.241	0.448
WTP/INC	Coefficient				1	0.504
	p value				-	0.000*
WTP	Coefficient					1
	p value					-

Where:

DLQI = total score of Dermatology Life Quality Index

Standard TTO = Quality of life measured by the standard Time Trade Off method

Daily TTO = Quality of life measured by the daily Time Trade Off method

WTP = Monthly Willingness To Pay (baht/month)

WTP/INC = Impact on quality of life measured by the Willingness To Pay method (monthly WTP/monthly income).

## Discussion

A previous study reported characteristics of melasma patients who were mostly female with an average age of about 40 years. Sun exposure was the most important predisposing factor for melasma [5,26]. This study demonstrated a similar result. A previous study reported that 20-70% of melasma patients had family members with melasma [5,27]. We found that 53.8% of patients had relatives with melasma, mostly their mother and/or sisters. The mean MASI score was 14.8, which was slightly higher than in the previous study [27]. About half of patients had educational level higher than bachelor's degree. Patients with higher education may pay more attention to their images; thereby seeking for the treatment of melasma. Most of patients received hydroquinone and sunscreen which were the main topical treatments for melasma in Thailand.

The DLQI has been used to determine the health-related quality of life in patients with skin problems and has been considered to be superior, in terms of reliability, to general (non-dermatological) measurements in dermatological patients [11,28]. Previous studies have reported a mean total DLQI score of 5.9-7.4 for psoriasis patients and 6.1-7.3 for atopic eczema patients [8,29]. Using the Thai version of the DLQI, Kulthanan et al. found that the mean overall DLQI score in Thai patients with psoriasis, acne, vitiligo and melasma was 12.9, 10.6, 8.8 and 6.0, respectively. The score for melasma patients was significantly higher than normal people and patients with viral warts, seborrheic keratosis, moles, and benign skin tumors [12]. The mean total DLQI score for melasma of 7.3 reported in the present study was slightly higher than that of the aforementioned study. This confirms that melasma has a significant adverse impact on the individuals' quality of life. A previous study demonstrated that melasma has a greater impact on the psychosocial rather than the physical aspects of a patient's life [30]. Similar to this study, the most common positively answered questions in the DLQI were associated with psychosocial aspects.

The health state utility was measured on a scale between 0 (death) and 1 (full health) by the TTO. In the current study, the mean health state utility of patients with melasma was 0.96 according to the standard TTO and 0.92 according to the daily TTO. Many studies on dermatology have used the standard TTO in patients with psoriasis [8,16,17,29], atopic dermatitis [8,16,29], and acne,[16] finding utility values of 0.56-0.93, 0.64-0.97, and 0.94, respectively. This implies that according to the standard TTO the utility of melasma individuals is as high as that of patients with psoriasis, atopic dermatitis, and acne.

Schmitt et al. reported that health utilities obtained using the standard TTO were independent of sex, age, occupational status, income, and objective disease severity [29]. Similarly, the present study demonstrated that utilities obtained by the standard TTO method were independent of sex, age, occupation, income, and MASI score. The results from previous studies about correlation between the DLQI and the standard TTO were inconclusive. Lundberg et al. reported a significant correlation between the DLQI score and utilities obtained by the standard TTO method [8]. In contrast, Schmitt et al. demonstrated that health utilities obtained by the standard TTO were independent of the impact on quality of life (DLQI) [29]. Our study demonstrated no significant correlation between DLQI and standard TTO. Thus, the correlation between the DLQI and the standard TTO remains uncertain.

Standard TTO has frequently been used to determine quality of life in diseases with physical disability and life-threatening characteristics [31,32]. Most dermatological diseases are nonlife- threatening and impair patients' psychosocial rather than physical aspects. The standard TTO may not be a good tool for measuring quality of life in patients with dermatological diseases, and should only be used in patients with more severe disease states (involving more physical aspects or those that are more life-threatening).

Schiffner et al. used the daily TTO to assess patients' quality of life. This method appeared to provide the patients with a realistic time schedule that was easier to imagine. Using the daily TTO method, patients with port-wine-stains (PWS) and psoriasis would exchange 1.2 and 2.8 hours per day for a cure, respectively [15,21]. The impact on the quality of life measured using the daily TTO in patients with melasma in the present study (1.9 hours) is between values obtained for PWS and psoriasis patients. In this study the daily TTO was significantly correlated with an economically inactive occupation, but not with total DLQI score. This implies that the daily TTO was influenced by socioeconomic factors rather than quality of life.

Measures of individuals' WTP is another approach with which to assess health-related quality of life [8]. Interpretation of WTP results is complicated because of the differences in therapies and currencies. In this study the WTP considered the most effective treatment of melasma instead of an imaginary treatment because no long-term cure is available. In Thailand, melasma patients have to pay out-of-pocket to receive treatment. Therefore, the WTP in this study should reflect the real impact of melasma on quality of life and provide an important basis upon which to expand treatment in the clinic as it is a real situation. Moreover, this WTP could be used in further cost-benefit analysis.

Lundberg et al. reported that patients' WTP for the cure of psoriasis and atopic eczema was about 9-14% and 8% of the average personal income, respectively. These values can be compared with the WTP for a cure in other diseases including asthma and acne [8]. Schiffner et al. reported that the WTP for a cure for PWS and psoriasis was 11.8% and 14% of monthly income, respectively [15,21]. Thus the impact on quality of life of melasma as measured by WTP was as high as that of atopic eczema. In 2007, the Thai National Economics and Social Development office demonstrated that 'non-poor' Thai people (income above poverty line, 1443 baht/capita/month) spent 31.9, 2.7, 13.8 and 18.6% of monthly income on food, clothes and footwear, housing and transportation, respectively. Thus patients in this study were willing to spend more on melasma treatment than on clothes and footwear.

Schmitt et al. reported a parallel increase in WTP with greater monthly income that was independent of sex, age, occupation, and objective disease severity [29]. Similarly, we found that the mean monthly WTP was significantly associated with monthly personal income but was independent of sex, age, occupation, and MASI score. Previous studies reported that the WTP was significantly correlated with the DLQI questions [8,29]. We also found that the mean monthly WTP and WTP in terms of relative monthly income were weakly (but statistically significantly) correlated with the total DLQI. Therefore, additional studies with larger sample sizes are needed to further confirm this association. And if the correlation is proved to exist, WTP may be the useful tool to measure dermatological patients' quality of life.

The correlation between individual demographic, clinical, and socioeconomic characteristics and quality of life based on the WTP and daily TTO demonstrates that younger patients and patients with economically inactive occupations have the worst quality of life due to melasma. Dermatologists should pay more attention to treating and caring for these groups of patients.

Limitations of this study included small sample size and only melasma patients visiting the clinic were evaluated, not the general population of people with melasma. Thus, there could be a lot of people who aren't bothered by melasma and who don't come to a dermatologist for an evaluation which will reflect the true WTP and TTO.

## Conclusion

The WTP method could be a useful tool for measuring the quality of life of patients with melasma.

## Competing interests

The authors declare that they have no competing interests.

## Authors' contributions

Drs. CL and RW had full access to all of the data in the study and takes responsibility for the integrity of data and the accuracy of the data analysis.

Study concept and design: Drs. CL and RW

Acquistition of data: Dr. CL

Analysis and interpretation of data: Drs. CL and RW

Drafting of the manuscript: Dr. CL

Critical revision of the manuscript for important intellectual content: Dr. RW

Statistical analysis: Dr. CL

Obtained funding: none

Administrative, technical, or material support: Dr. WM

Study supervision: Drs. WM and KK

All authors read and approved the final manuscript.

## Pre-publication history

The pre-publication history for this paper can be accessed here:

http://www.biomedcentral.com/1471-5945/11/16/prepub
